# Electron Microscopic Recording of the Power and Recovery Strokes of Individual Myosin Heads Coupled with ATP Hydrolysis: Facts and Implications

**DOI:** 10.3390/ijms19051368

**Published:** 2018-05-04

**Authors:** Haruo Sugi, Shigeru Chaen, Tsuyoshi Akimoto

**Affiliations:** 1 Department of Physiology, School of Medicine, Teikyo University, Tokyo, Japan; microscopy@sanko-it.com; 2 Department of Integrated Sciences in Physics and Biology, College of Humanities and Science, Nihon University, Tokyo, Japan; chaen.shigeru@nihon-u.ac.jp

**Keywords:** myosin head power stroke, myosin head recovery stroke, muscle contraction, gas environmental chamber, myosin head neutral configuration

## Abstract

The most straightforward way to get information on the performance of individual myosin heads producing muscle contraction may be to record their movement, coupled with ATP hydrolysis, electron-microscopically using the gas environmental chamber (EC). The EC enables us to visualize and record ATP-induced myosin head movement in hydrated skeletal muscle myosin filaments. When actin filaments are absent, myosin heads fluctuate around a definite neutral position, so that their time-averaged mean position remains unchanged. On application of ATP, myosin heads are found to move away from, but not towards, the bare region, indicating that myosin heads perform a recovery stroke (average amplitude, 6 nm). After exhaustion of ATP, myosin heads return to their neutral position. In the actin–myosin filament mixture, myosin heads form rigor actin myosin linkages, and on application of ATP, they perform a power stroke by stretching adjacent elastic structures because of a limited amount of applied ATP ≤ 10 µM. The average amplitude of the power stroke is 3.3 nm and 2.5 nm at the distal and the proximal regions of the myosin head catalytic domain (CAD), respectively. The power stroke amplitude increases appreciably at low ionic strength, which is known to enhance Ca^2+^-activated force in muscle. In both the power and recovery strokes, myosin heads return to their neutral position after exhaustion of ATP.

## 1. Introduction

In 1954, H.E. Huxley and Hanson [1] made a monumental discovery that muscle contraction is produced by the sliding between actin and myosin filaments, which is believed to be caused by cyclic attachment and detachment between myosin heads extending from myosin filaments and the corresponding myosin-binding sites in actin filaments. Muscle is regarded as a machine to convert chemical energy of ATP hydrolysis into mechanical work.

**Figure 1:**
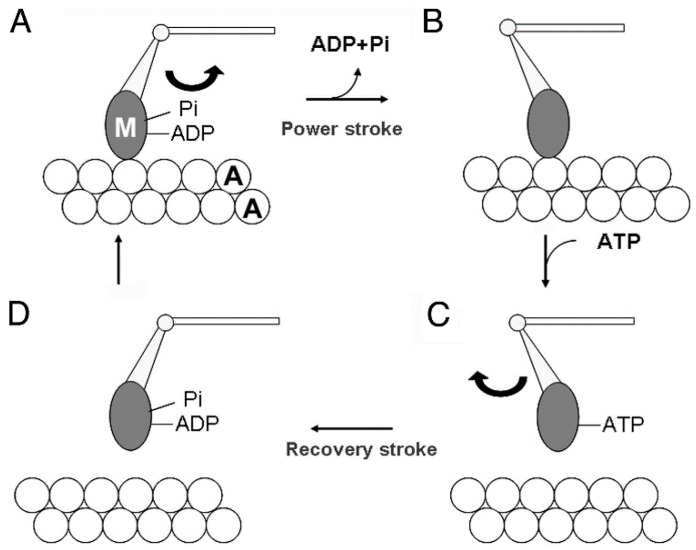
Diagram showing the attachment-detachment cycle between myosin head (M) extending from myosin filament and actin filament (**A**), coupled with ATP hydrolysis. For further explanation, see text. From Sugi et al., [6].

Figure 1 illustrates the most plausible sequence of events taking place in contracting muscle, based on actomyosin ATPase reaction steps in solution [2]. The myosin head (M) in the form of M–ADP–Pi first binds with an actin filament (A) to form A–M–ADP–Pi (A), and then changes its configuration to perform the power stroke, associated with the reaction, A–M–ADP–Pi → A–M + ADP + Pi, to produce unitary sliding between the actin and myosin filaments (A to B). After completion of the power stroke, M remains bound to A until the next ATP comes to bind to it (B). On binding with ATP, M detaches from A (C), and performs a recovery stroke associated with the reaction, M–ATP → M–ADP–Pi, to return to its original configuration (C to D). In order to repeat cyclic changes in configuration, the power and recovery strokes should be the same in amplitude, and opposite in direction. Despite extensive studies on contracting muscle using methods of muscle mechanics [3], chemical probes attached to myosin heads [4], and time-resolved X-ray diffraction [5], it has not been possible to clearly prove and characterize the myosin head power and recovery strokes mainly because of the asynchronous nature of myosin head movement.

The most straightforward means to study the power and recovery strokes in individual myosin heads is to record their movement in response to ATP electron microscopically. It has been a dream of life scientists to observe living organisms under an electron microscope, and a number of attempts have been made to study living microorganisms in aqueous environment electron-microscopically. Such attempts were, however unsuccessful because of electron beam damage that destroyed cell functions [7]. Meanwhile, it seemed possible to study dynamic structural changes of biomolecules, such as actin and myosin, using a gas environmental chamber (EC), in which biological specimens are insulated from the high vacuum of electron microscopes and are kept in aqueous solution. Fortunately, we have been able to use an EC system, developed by the late Professor Akira Fukami of Nihon University with the aid of Japan Electron Optics Laboratory, Ltd. (JEOL, Akishima, Tokyo, Japan), and we succeeded in visualizing and recording ATP-induced myosin head movements in hydrated, living myosin filaments electron microscopically. In this article, we only describe the results obtained from actin and myosin filaments of rabbit psoas muscle fibers, though we have also made preliminary experiments using myosin–paramyosin core complex filaments [8].

This section may be divided by subheadings. It should provide a concise and precise description of the experimental results, their interpretation, as well as the experimental conclusions that can be drawn.

## 2. Experiments with the EC System

### 2.1. The EC System

**Figure 2:**
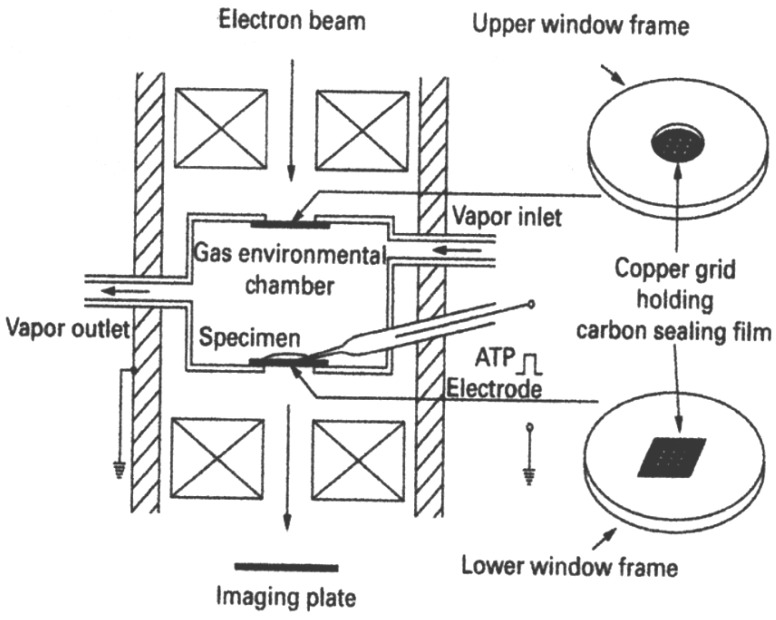
Diagram of the EC. ATP is applied to the specimen iontophoretically by passing a current through the ATP electrode, containing 100 mM ATP. For further explanation, see text. From Sugi et al., [8].

As illustrated in Figure 2, the EC used was a cylinder (inner diameter, 2.0 mm; depth, 0.8 mm) with upper and lower windows to pass the electron beam. Each window is covered with a thin carbon sealing film (thickness, 15–20 nm) held on a copper grid with nine apertures (diameter, 0.1 mm). The carbon film could resist against pressure difference up to 1 atmosphere. The specimen placed on the lower carbon film was kept wet by a constantly circulating water vapor (pressure, 60–80 torr; temperature, 26–28 °C). The EC contained a glass microelectrode filled with 100 mM ATP to apply ATP to the specimen iontophoretically by passing a current pulse (intensity, 10 nA; duration, 1 s) through the electrode. The ATP concentration around the specimen was calculated to be <10 µM. The EC was used in a 200 kV transmission electron microscope (JEM 2000EX; JEOL). The specimen images (magnification, 10,000×) were recorded with the imaging plate system (PIX system, JEOL). The total incident electron dose was limited below 5 × 10^−4^ C/cm^2^ to avoid electron beam damage to the specimen [8]. Further details of the EC system, including the carbon sealing film, have been described elsewhere [6,8,9,10,11,12].

### 2.2. Position Marking of Individual Myosin Heads

**Figure 3:**
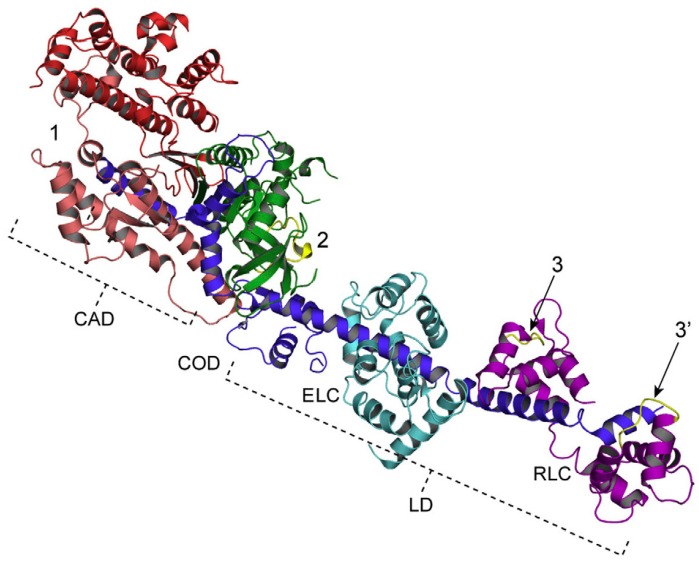
Myosin head structure showing approximate regions of attachment of antibodies 1, 2 and 3, indicated by numbers 1, 2 and 3 and 3’, respectively. The catalytic domain (CAD) consists of 25 K (green), 50 K (red), and 20 K (dark blue) fragments of myosin heavy chain, while the lever arm domain (LD) consists of the rest of the 20 K fragment and essential (ELC, light blue) and regulatory (RLC, magenta) light chains. The CAD and the LD are connected by the converter domain (COD). Location of peptides around Lys83, and the location of two peptides (Met58~Ala70 and Leu106~Phe120) in the LD are colored yellow. From Minoda et al., [14].

**Figure 4:**
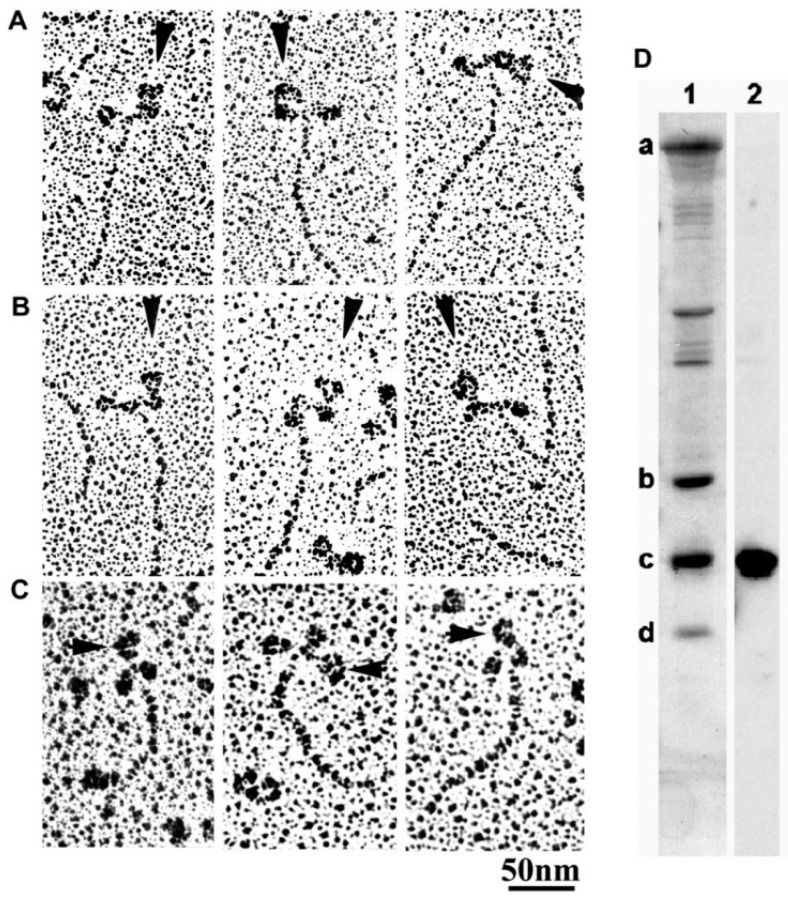
(**A**–**C**) A gallery of electron microscopic images of rotary shadowed antibody 1, 2 or 3 (IgG)–myosin complex. IgG molecules are indicated by arrowheads. Panels A and B are provided from the published paper [13]. (**D**) Binding specificity of antibody 3 for myosin regulatory light chain. Proteins in myosin sample are separated on SDS-PAGE, and transferred onto a PVDF membrane. In lane 1, all myosin subunit and light chains are visualized by Coomassie staining, while in lane 2, regulatory light chain is specifically visualized by Western blot using antibody 3. From Minoda et al., [14].

In vertebrate skeletal muscle, a myosin head consists of an approximately oval-shaped catalytic domain (CAD) and a rod-shaped lever arm domain (LD), which are connected via small converter domain (COD). The myosin head LD is connected to myosin subfragment-2 (S-2) extending from the myosin filament backbone (Figure 3). It is absolutely necessary to position-mark individual myosin heads in myosin filaments, mounted in the EC in an unstained condition. For this purpose, we used three different kinds of monoclonal antibody, which will hereafter be called antibodies 1, 2, and 3. Antibody 1 attaches to the junctional peptide between the 50 K and 20 K segments of the myosin heavy chain in the myosin head CAD [13]. Antibody 2 attaches to reactive lysine residue in the myosin head COD [13]. Antibody 3 attaches to two light chains in the myosin head LD [14]. In Figure 3, approximate regions of attachment of antibodies 1, 2, and 3 are indicated by numbers 1, 2, and 3 and 3’, respectively. As shown in Figure 4, antibodies 1 and 2 (IgG) were confirmed to actually attach to myosin head CAD regions (A and B), while antibody 3 molecules were seen to attach the head–rod junction, i.e., myosin head LD regions (C).

**Figure 5:**
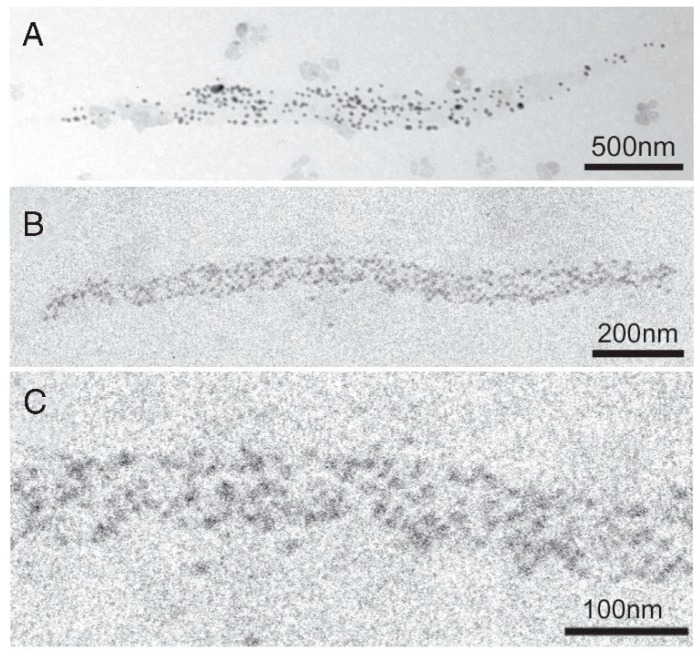
(**A**,**B**) Typical IP records of synthetic myosin filaments with a number of gold particles attached to individual myosin heads. The myosin filaments are spindle-shaped with two tapered ends. (**C**) Enlarged IP record showing part of the myosin filament in A. Each gold particle consists of 20–50 dark pixels. From Sugi et al., [6].

White male rabbits (2–2.5 kg) were killed by injection of sodium pentobarbital (50 mg/kg) into the ear vain, and psoas muscles were dissected from the animals. Actin (F-actin) and myosin were prepared from the psoas muscle. The specimens used for the experiments described in this section were synthetic myosin filaments consisting of a myosin–myosin rod mixture, prepared by mixing myosin and myosin rod at a molar ratio of 1:1 and then slowly polymerizing them by dialysis against a low ionic strength solution. Further details of the methods have been described elsewhere [6,9,10]. Figure 5 shows typical IP images of the synthetic bipolar myosin filaments, in which individual myosin heads are position-marked by colloidal gold particles (diameter, 20 nm; coated with protein A serving as a paste) via antibody 1 (A, B). The filament diameter at the middle was 50–250 nm, while the filament length was 0.1–3.5 µm. The image of each gold particle consisted of 20–50 dark pixels, with a wide range of variation (C) [6,9,10].

### 2.3. Recording and Analysis of the ATP-Induced Myosin Head Movement

Filament images were recorded on the imaging plate under a magnification of 10,000× (exposure time, 0.1 s). Because of the need to limit the total incident electron dose, the same filaments could only be recorded 2 to 4 times. In the above condition, the pixel size on the imaging plate (IP) was 2.5 × 2.5 nm, and the average number of electrons reaching each pixel during the exposure time was ~10. Reflecting the electron statistics, each gold particle image consisted of 20–50 pixels. After application of appropriate contrast enhancement or binarization procedures to obtain a clear particle position, particles suitable for analysis were selected [6,9,10].

The centre of mass positions for each selected particle was determined as the coordinates (two significant figures) within a single pixel where the centre of mass position was located. These coordinates, representing the position of individual myosin heads, were compared between two different IP records of the same filaments. The absolute coordinates common to the two IP records were obtained based on the position of natural markers (bright spots on the IP film). The distance D between the two centre of mass positions (with the coordinates, X1, Y1, and X2, Y2, respectively) was calculated as D = √(X1 − X2)^2^ + (Y1 − Y2)^2^ [6,9,10].

## 3. Myosin Head Movements in Response to ATP

The experiment with the EC system has the following advantages over other methods with respect to detection of myosin head movements during muscle contraction. (1) Unlike all other methods to detect myosin head movement, it makes it possible to visualize and record individual myosin heads under a high electron microscopic magnification; (2) In the myosin filaments used, myosin heads extend from the myosin filament backbone in the same manner as in native myosin filaments in muscle; (3) In the in vitro motility assay experiment, the mode of fixation and orientation of myosin molecules or their fragments, heavy meromyosin or myosin head, is not clear at all to make the interpretation of the results ambiguous; (4) Using the three different site-directed antibodies, it is possible to record ATP-induced movement at three different regions within a single myosin head.

### 3.1. Stability in the Position of Myosin Heads in the Absence of ATP

**Figure 6:**
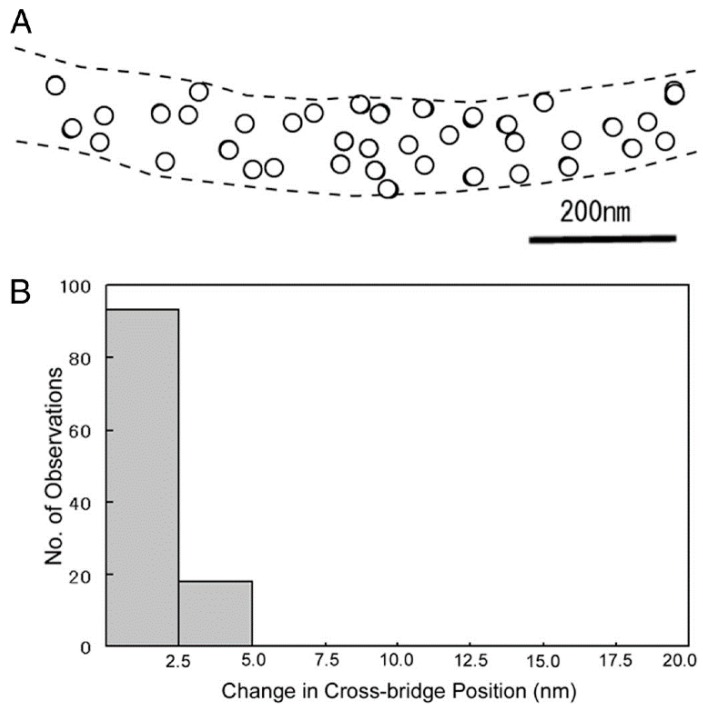
Stability of time-averaged myosin head mean position in the absence of ATP. (**A**) Difference in the myosin head position between two IP records of the same myosin filament on the common coordinates. Open and filled circles (diameter, 20 nm) are drawn around the center of mass position of each gold particle in the first and the second IP records, respectively. Note that filled circles are barely visible because of nearly complete overlap of open and filled circles. (**B**) Histogram of distance between the two center of mass positions of the same particle in the first and the second records. From Sugi et al., [6].

As shown in Figure 6, the position of individual myosin heads in myosin filaments remain unchanged with time in the absence of ATP, indicating that the myosin head position, time-averaged over 0.1 s (exposure time of filament image to the IP film), remains almost unchanged with time. This finding is accounted for as being due to thermal fluctuation of each myosin head taking place around a definite equilibrium position [8], as predicted by Huxley [15]. The equilibrium position will hereafter be called the “neutral position” to emphasize that myosin head thermal fluctuations are three dimensional. Evidence will be presented later in this article that, in the neutral position, myosin heads take an average configuration perpendicular to the myosin head backbone. The stability of this myosin head position gives a favorable condition to record the movement produced by ATP.

### 3.2. Amplitude and Direction of ATP-induced Myosin Head Movement in the Absence of Action Filament

**Figure 7:**
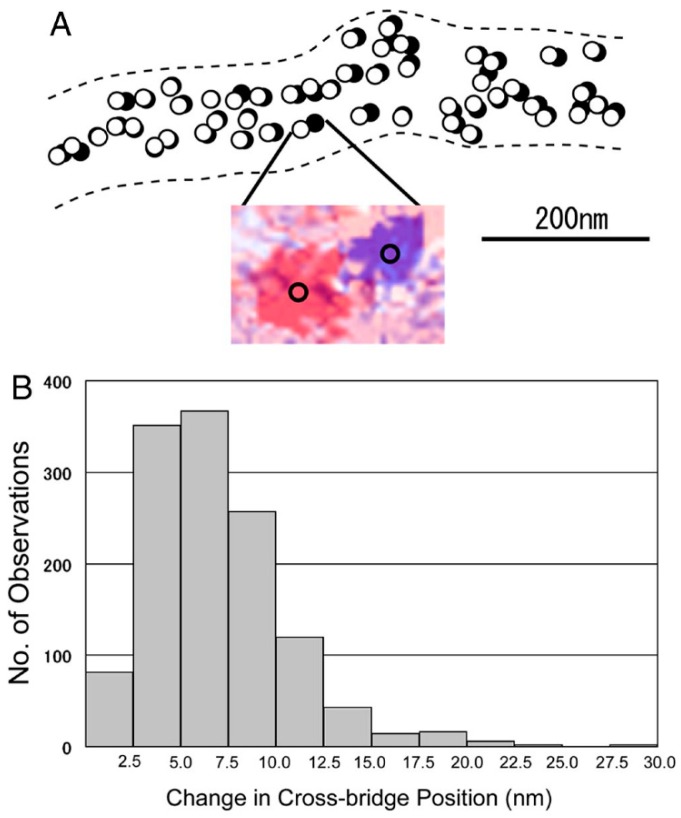
ATP-induced myosin head movement in the absence of actin filaments. (**A**) Comparison of the myosin head position between the two IP records, taken before and after ATP application, respectively. Open and filled circles (diameter, 20 nm) are drawn around the centre of mass position of the same particles before and after ATP application, respectively. (Inset) An example of superimposed IP records showing the change in position of the same particle, which are colored red (before ATP application) and blue (after ATP application). The center of mass position for each particle image is located at the center of the circle on the particle image. (**B**) Histogram of amplitude distribution of ATP-induced myosin head movement. From Sugi et al., [6].

**Figure 8:**
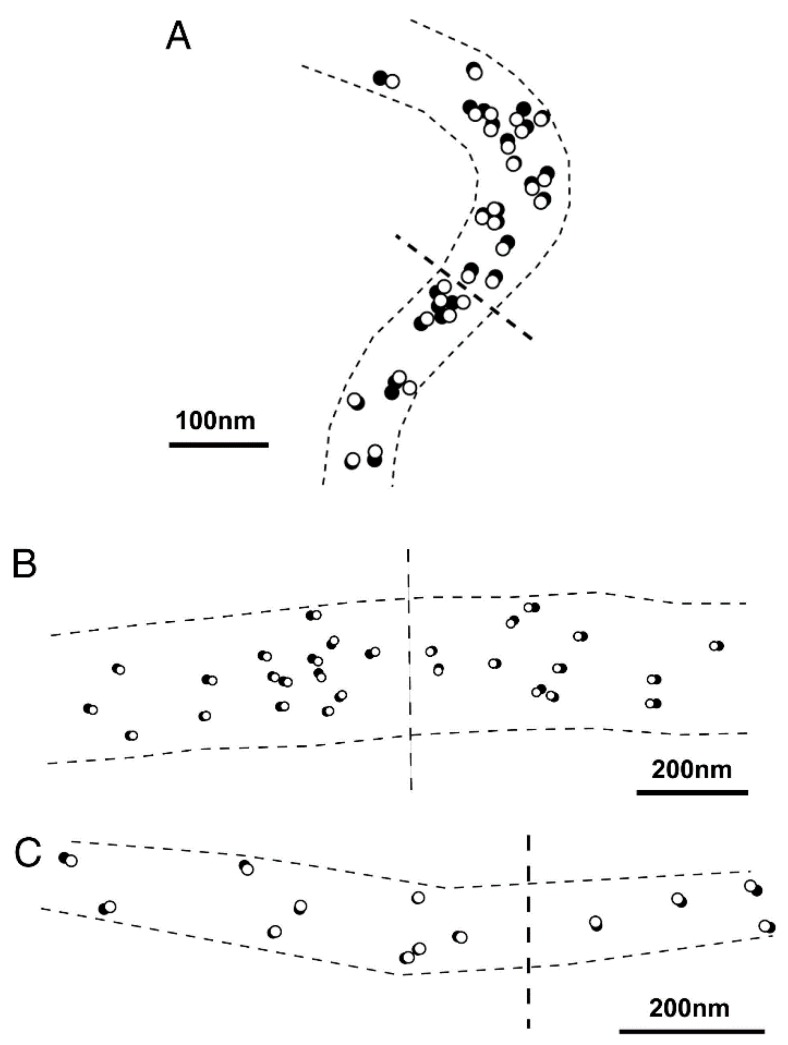
(**A**−**C**) Examples of IP records showing the ATP-induced myosin head movement at both sides of myosin filament bare region, across which myosin head polarity is reversed. Open and filled circles (diameter, 20 nm) are drawn around the center of mass positions of the same particles in the IP records, taken before and after ATP application, respectively. Note that myosin heads move away from the bare region. Approximate location of the bare region is indicated by broken lines across the center of myosin filament. From Sugi et al., [6].

Based on the stability of the neutral myosin head position, ATP-induced myosin head movement can be recorded by comparing two IP records of the same filament, one taken before and the other after application of ATP [8]. A typical result is shown in Figure 7. In response to ATP, individual myosin heads move parallel to the filament axis in one direction with an average amplitude of ~7 nm. In several experiments, we were able to record the ATP-induced myosin head movement on both sides of the bare region, located at the centre of myosin filaments. As can be seen in Figure 8, individual myosin heads move away from, but not towards, the bare region across which the polarity of myosin head is reversed. This finding constitutes the first direct demonstration of a myosin head recovery stroke, since myosin heads in the absence of actin filaments, i.e., detached from actin filaments, are believed to perform a recovery stroke when ATP binds with them (Figure 1C,D). This finding is also taken to indicate that myosin heads can perform this recovery stroke without being guided by actin filaments. When iontophoretically applied ATP reaches myosin heads (M), it binds with M to form a complex associated with the reaction, M + ATP → M–ATP → M–ADP-Pi. As M–ADP–Pi has long lifetimes > 10 s [2], myosin heads after completion of recovery stroke can be clearly recorded on the IP despite the limited time resolution (0.1 s) of IP recording.

**Figure 9:**
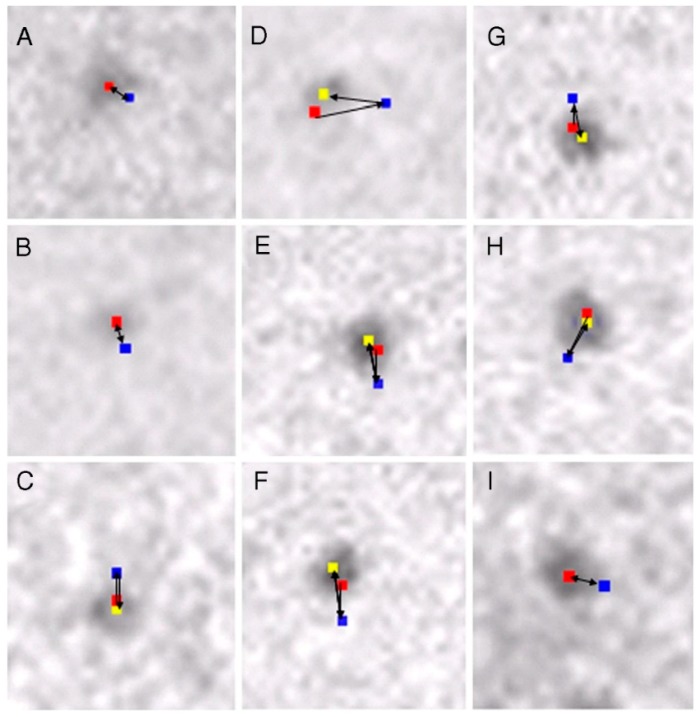
(**A**−**I**) Examples of sequential changes in position of 9 different pixels (each 2.5 × 2.5 nm), where the center of mass positions of corresponding different particles are located. In each frame, the pixel positions were recorded 3 times, i.e., before ATP application, during ATP application, and after complete exhaustion of applied ATP. The changes in the pixel position in the first (red), second (blue), and the third record (yellow) represent the changes in the myosin head position before ATP application, during ATP application, and after complete exhaustion of ATP. Direction of each myosin head movement is indicated by arrows. Note that myosin heads return towards their initial position after exhaustion of ATP. From Sugi et al., [6].

We took IP records of the same filament three times: (1) before ATP application; (2) during ATP application, and (3) after complete exhaustion of applied ATP, which was facilitated by adding hexokinase and D-glucose to the experimental solution [16]. As shown in Figure 9, myosin heads, which had performed recovery stroke, returned towards their initial (i.e., neutral) position (or configuration) after complete exhaustion of applied ATP. The variability of distance in pixel movement as well as the incomplete return of some myosin heads to the original position may be caused by the movement of adjacent non position-marked myosin heads. The results shown in Figure 9 may therefore be taken to indicate that myosin heads can perform movement in the direction of the power stroke, when they return to the initial position. This point will be discussed later in connection with myosin head neutral configuration.

**Figure 10:**
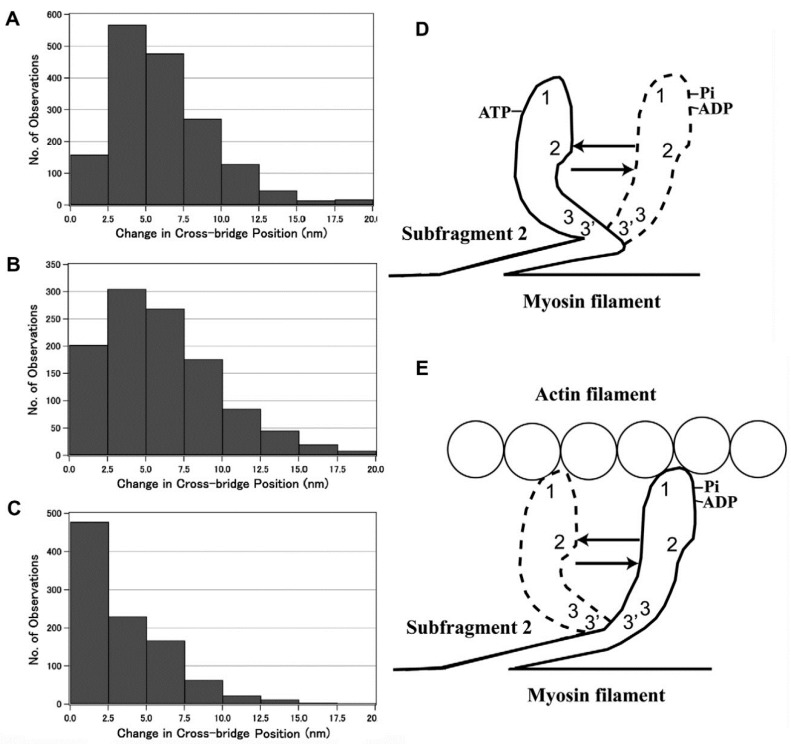
(**A**–**C**) Histograms showing the amplitude distribution of ATP-induced myosin head recovery stroke at the distal (**A**), the proximal (**B**) regions of myosin head CAD, and at the two regulatory light chains located at the proximal region of myosin head LD. (**D**,**E**) Diagrams illustrating the ATP-induced changes in myosin head configuration in the absence (**D**) and in the presence (**E**) of actin filaments, based on the histograms shown in (**A**–**C**). From Minoda et al., [14].

We also recorded the ATP-induced movement at three different regions within a myosin head using antibodies 1, 2, and 3, attaching to the distal and the proximal regions of myosin head CAD, and myosin head LD, respectively (Figure 10) [14]. The amplitude of movement was the same in the distal region (6.14 ± 0.09 nm, mean ± SEM, *n* = 1692) and the proximal region (6.14 ± 0.22 nm, *n* = 1112) of myosin head CAD, while that at the myosin head LD region was much smaller (3.55 ± 0.11 nm, *n* = 981).

### 3.3. Two Different Modes of Myosin Head Power Stroke

**Figure 11:**
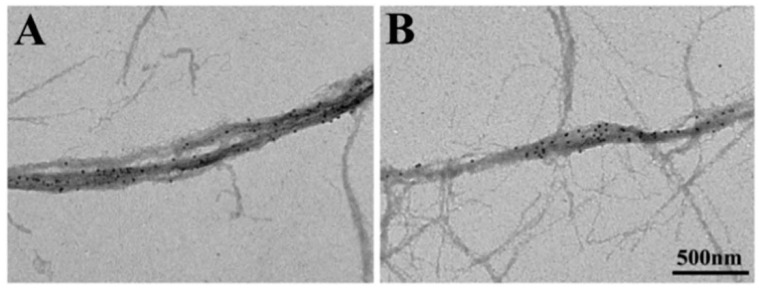
Conventional electron micrographs showing part of actin and myosin filament mixture. Myosin heads were position-marked with antibody 1 (**left**) and antibody 2 (**right**). Note that thick myosin filaments are surrounded by thin actin filaments. From Sugi et al., [10].

**Figure 12:**
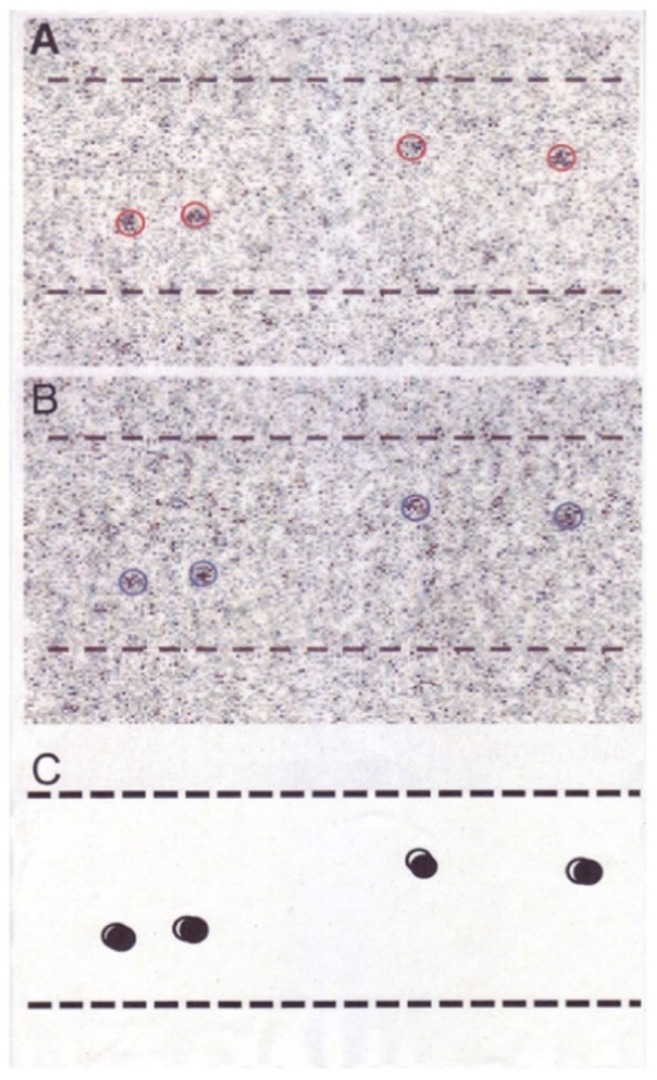
An example of a pair of IP records of the same myosin filament mixture (**A**,**B**). Records A and B are taken before and after ATP application, respectively. Circles (diameter, 20 nm) are drawn around the center of mass positions of individual gold particle images, consisting of a number of dark pixels. (**C**) Diagram showing ATP-induced changes in position of gold particles attached to individual myosin heads with antibody 1. Open and filled circles (diameter, 20 nm) are drawn around the center of mass positions of the same particles before and after ATP application, respectively. Broken lines indicate approximate contour of myosin filaments. From Sugi et al., [10].

**Figure 13:**
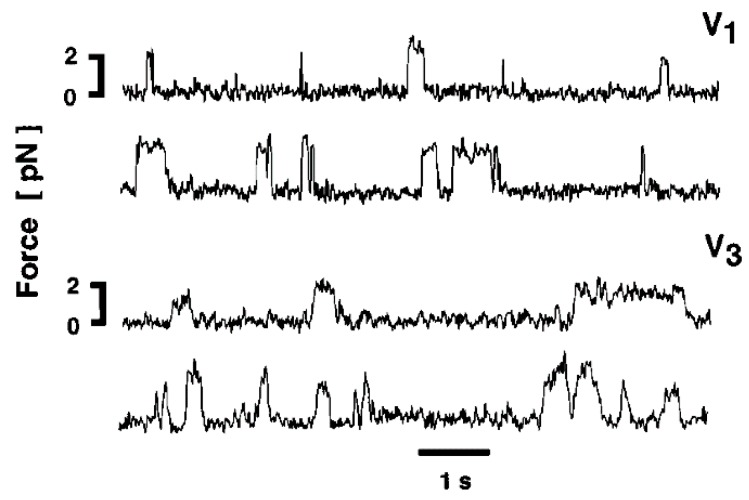
Records of force transients obtained for V1 and for V3 cardiac myosin isoforms using a high trap stiffness of the optical trap experiment. Vertical deflections indicate movement of the trap position, i.e., transient force generation of a single myosin head fixed on a bead. From Sugiura et al., [19].

We also performed experiments to record ATP-induced myosin head power stroke using the actin and myosin filament mixture, in which spindle-shaped synthetic myosin filaments are surrounded by actin filaments (Figure 11) [10]. Before ATP application, myosin heads form tight rigor linkages with actin filaments. This was confirmed by the stability of the myosin head position, which was much more pronounced compared to that when actin filaments were absent (Figure 6). Immediately after mixing of actin and myosin filaments, some myosin heads form linkages with actin in a distorted, tension-bearing configuration. Due to the finite lifetimes of the rigor linkages [17], such linkages are gradually broken, and myosin heads detaching from actin first return to their neutral configuration and then again form rigor linkages with actin located just opposite to them. Since the experiments were made at ~30 min after mixing of actin and myosin filaments, almost all myosin heads may be expected to have formed rigor linkages at their neutral positions, where they exert no force. Consequently, as soon as myosin heads bind with applied ATP, they detach from actin to form the complex M–ADP–Pi at their neutral position, and they then start binding with actin filament to perform the power stroke. A typical result showing the ATP-induced changes in myosin head position is presented in Figure 12. In response to ATP, individual myosin heads exhibited small but distinct changes in their position, despite the limited time resolution of the IP recording (0.1 s). The reason for this is the limited ATP concentration (<10 µM) around myosin filaments; after completion of the power stroke, myosin heads remain attached to an actin filament until the next ATP comes to bind to it. This is for 0.5 to 1 s or more, as indicated in the force spikes recorded in the optical trap experiment at a low ATP concentration <10 µM [18,19] (Figure 13). It can be seen that a single myosin head in the optical trap exhibits force spikes with peaks (tension generating state) of >0.2 s duration with an interval between peaks up to more than several s. The same situation applies to myosin heads in the EC experiments.

**Figure 14:**
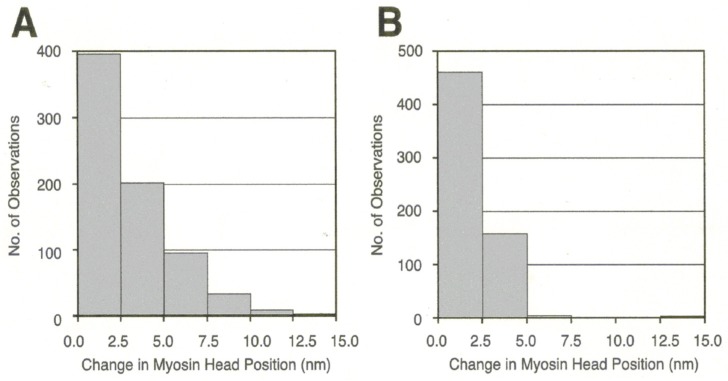
Histograms showing the amplitude distribution of ATP-induced myosin head power stroke at standard KCl concentration (120 mM). Individual myosin heads were position-marked with antibody 1 at the distal region of myosin head CAD in (**A**), and with antibody 2 at the proximal region of myosin head CAD in (**B**). From Sugi et al., [10].

**Figure 15:**
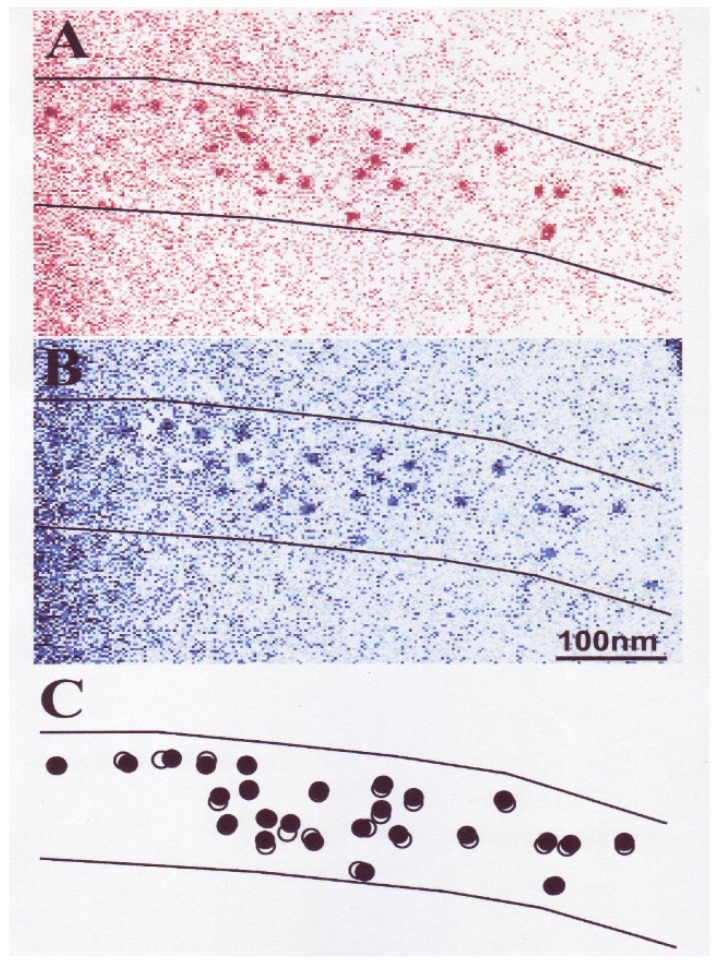
(**A**,**B**) A pair of IP records of the same myosin filament in the filament mixture. Records A and B were taken before and after ATP application, respectively. Recordings were made at low KCL concentration (20 mM). (**C**) diagram showing ATP-induced changes in position of gold particles attached to individual myosin heads with antibody 2. Open and filled circles (diameter, 20 nm) were drawn around the center of mass positions of the same particles before and after ATP application, respectively. From Sugi et al., [10].

**Figure 16:**
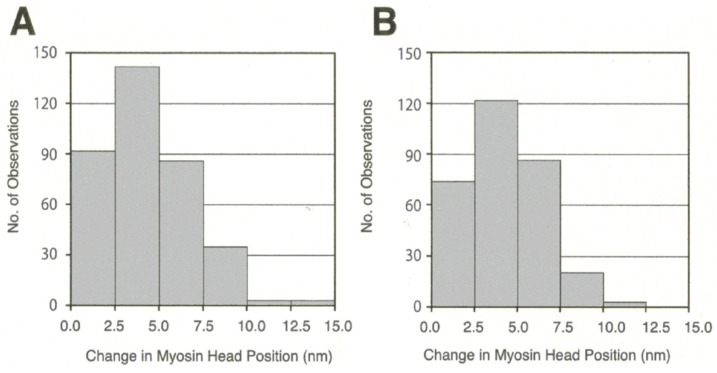
Histograms showing the amplitude distribution of ATP-induced myosin head power stroke at external KCl concentration of 20 mM. Individual myosin heads were position-marked with antibody 1 at the distal region of myosin head CAD in (**A**), and with antibody 2 at the proximal region of myosin head CAD in (**B**). From Sugi et al., [10].

In the actin–myosin filament mixture, myosin heads perform a power stroke for a limited distance because only a very small fraction of myosin heads can be activated with the small concentration of ATP applied (<10 µM). In this condition, myosin heads can only perform a power stroke by stretching adjacent elastic structures without producing gross actin–myosin filament sliding. The above condition resembles the nominally “isometric” condition, in which a muscle is activated with both ends fixed in position. Figure 14 presents histograms showing the amplitude distribution of the ATP-induced myosin head power stroke at the distal (A) and the proximal (B) regions of the myosin head CAD [10]. The power stroke amplitude in the isometric condition was significantly larger at the distal region (3.3 ± 0.2 nm, mean ± SD, *n* = 732) than at the proximal region (2.5 ± 0.1 nm, mean ± SD, *n* = 613) (t test, *p* < 0.01). Meanwhile, if the ionic strength of experimental solution was lowered from 125 to ≥20 mM, the average amplitude of the myosin head power stroke increased to 4.4 ± 0.1 nm (mean ± SD, *n* = 361) at the distal region, and 4.3 ± 0.2 nm (*n* = 305) at the proximal region of the myosin head CAD [10]. As was the case in the myosin head recovery stroke in the absence of actin filaments [6], the amplitude of movement was not significantly different between the distal and the proximal regions of myosin head CAD (Figure 15 and Figure 16).

**Figure 17:**
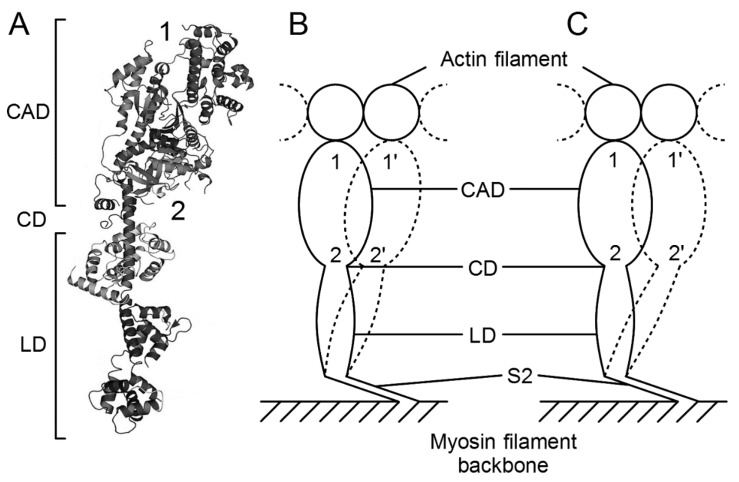
Two different modes of myosin head power stroke depending on experimental conditions. (**A**) Diagram showing myosin head structure consisting of catalytic domain (CAD), converter domain (COD), and lever arm domain (LD). Approximate attachment regions of antibodies 1 and 2 are indicated by numbers 1 and 2, respectively. (**B**) The mode of myosin head power stroke in the nearly isometric condition, in which gross myofilament sliding does not take palace (external KCl, 120 mM). Note that the amplitude of power stroke is larger at the distal region than at the proximal region in the myosin head CAD. (**C**) The mode of myosin head power stroke in the nearly isometric condition (external KCl, 20 mM). Note that the amplitude of myosin head power stroke is similar at both distal and proximal regions of myosin head CAD. From Sugi et al., [10].

Figure 17 summarizes the results on the two different modes of the myosin head power stroke depending on the experimental conditions [10]. In the standard ionic strength, the amplitude of the power stroke was larger at the distal region than at the proximal region of the myosin head CAD, while at low ionic strength the amplitude of power stroke was the same at both the distal and the proximal regions. Consequently, at the end of the power stroke in the nominally isometric condition, the configuration of the myosin head CAD is oblique to the axis of actin and myosin filaments at the standard ionic strength, while at low ionic strength the myosin heads do not change their configuration perpendicular to the filament axis. As already mentioned, it is a great advantage of the EC experiments that we can record the amplitude of myosin head motion at different regions within a single myosin head.

**Figure 18:**
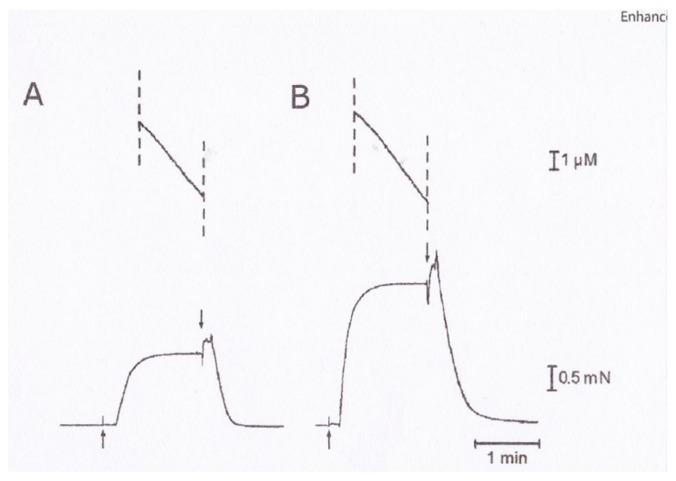
Simultaneous recordings of MgATPase activity (upper traces) and isometric force (lower traces) in a maximally Ca^2+^-activated skinned skeletal muscle fiber. Records (**A**) and (**B**) were obtained at the standard ionic strength and at low ionic strength, respectively. Note that the slope of the ATPase records remains the same in (A) and (B), while isometric force is two times larger in B than in A. From Sugi et al., [20].

In a separate study, we have found that, at low ionic strength, the maximum Ca^2+^-activated isometric force in skinned skeletal muscle fibers increases ~twofold without changing the MgATPase activity (Figure 18), indicating that the force generated by individual myosin heads increases ~twofold at low ionic strength [20]. This finding is completely in accord with the increase of power stroke amplitude at low ionic strength (Figure 16 and Figure 17); when individual myosin heads perform the power stroke by stretching adjacent elastic structures, the power stroke amplitude increases with increasing force generated by individual myosin heads. These results therefore support that the properties of myosin heads revealed by the EC experiments give direct information about those of myosin heads in relaxed and contracting muscles.

### 3.4. Fundamental Properties Incorporated in Myosin Heads as Revealed by EC Experiments and Freeze-Fracture Studies on Skinned Muscle Fibers

**Figure 19:**
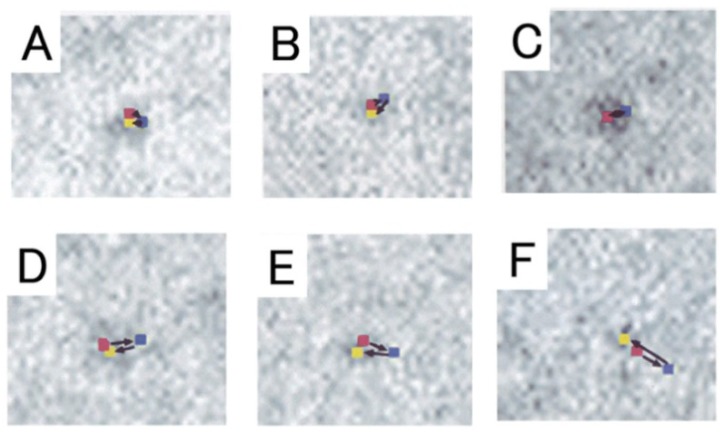
Sequential changes in position of 6 different pixels (each 2.5 × 2.5 nm) where the center of mass positions of corresponding particles are located. In each frame, pixel positions are recorded three times, i.e., before ATP application (red), after ATP application (blue), and after complete exhaustion of applied ATP (yellow). Direction of pixel movement, i.e., direction of myosin head movement, is indicated by arrows. Frames (**A**,**C**,**D**) were obtained from myosin heads position marked with antibody 1, while frames (**B**,**E**,**F**) were obtained from myosin heads position-marked with antibody 2. From Sugi et al., [10].

We also took IP records of the same myosin filament three times, i.e., (1) before ATP application; (2) during ATP application; and (3) after complete exhaustion of ATP. As has been the case for the myosin head recovery stroke, myosin heads always returned towards their initial position before ATP application (Figure 19). The return of myosin heads to the initial position was complete after small amplitude power strokes, and incomplete for large amplitude power strokes. Since the incomplete return may be caused by distortion of actin–myosin filament network caused by movements of adjacent non position-marked myosin heads, individual myosin heads may be regarded to exhibit only two definite positions in the IP records. As already mentioned, the same explanation may apply to the recovery stroke in the absence of actin filament (Figure 9).

**Figure 20:**
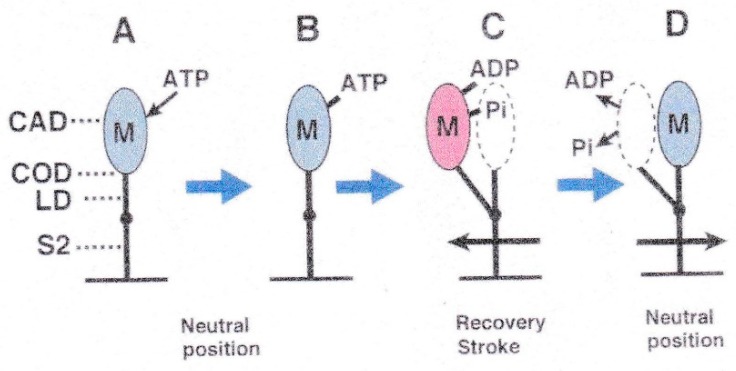
Diagrams illustrating the sequence of ATP-induced myosin head (M) recovery stroke in the absence of actin filaments. (**A**) M fluctuates around its neutral configuration before application of ATP. (**A** to **B**) On ATP application, ATP binds with M. (**B** to **C**) M then hydrolyses ATP to form complex M–ADP–Pi (charged-up state), and performs recovery stroke in the direction opposite to that of power stroke. (**D**) M releases ADP and Pi, and returns to its neutral configuration. M in charged-up state and non-charged-up state are colored red and blue, respectively, in this and Figure 21.

These results may therefore be taken to indicate rapid transitions among the three myosin head configurations, i.e., the neutral, post-power stroke, and post-recovery stroke configurations. Figure 20 shows diagrams which explain the results of the EC experiments without actin filaments given in Figure 6, Figure 7, Figure 8, Figure 9, Figure 10 and Figure 11 [14,21]. For the sake of simplicity, myosin head CAD (including COD) is shown as an oval, while the myosin head LD and myosin S-2 are shown as solid lines extending from the myosin filament backbone (bottom horizontal line), so that changes in myosin head configuration are shown as rotations of the myosin head LD around the LD–S-2 junction (indicated by a small black circle on the straight line). The myosin head CAD is assumed to be always at right angles with actin filament. Myosin heads (M) in the form of M and M–ATP are colored blue, while myosin heads in the form of M–ADP–Pi are colored red, indicating that they retain chemical energy derived from ATP hydrolysis; namely, they are in a “charged-up” state.

The ATP applied in the absence of actin filaments binds with M to form M–ATP (A to B). Then, M hydrolyzes bound ATP into ADP and Pi, while it performs a recovery stroke in the direction away from the bare region located at the centre of myosin filaments (Figure 8, B to C) [6]. This clearly indicate that, even in the absence of actin filaments, myosin heads have a fundamental property to determine the direction of their stroke without being guided by actin filaments. After completion of the recovery stroke, M releases ADP and Pi and rapidly returns to its neutral configuration (Figure 8D), while the energy obtained from ATP hydrolysis is dissipated as heat. The result is that, despite the limited time resolution (0.1 s) of the IP recording, only two definite myosin head positions are recorded for both the power and recovery strokes (Figure 9 and Figure 19), can be understood by the explanation stated above. 

**Figure 21:**
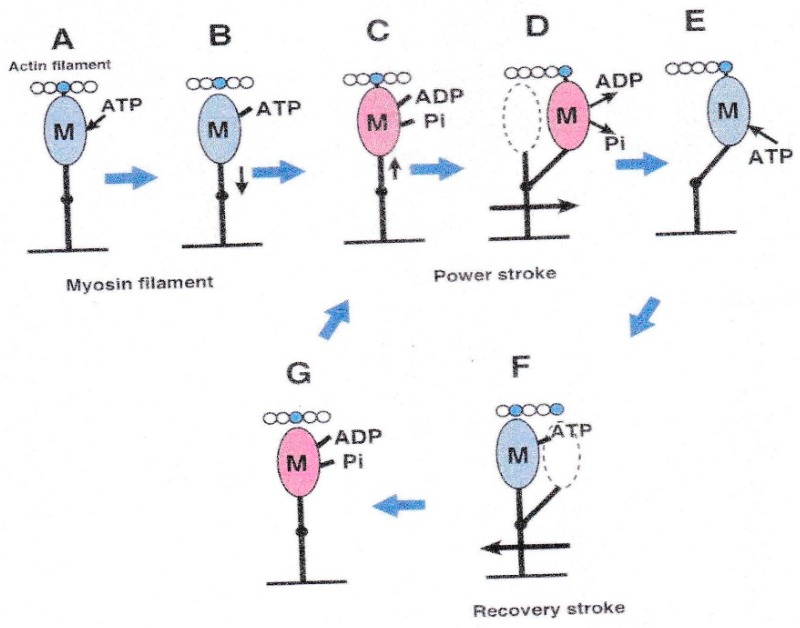
Diagrams illustrating the sequence of ATP-induced Power stroke in the actin–myosin filament mixture. (**A**) Myosin head (M), at its neutral position, forms rigor linkage with the actin filament. (**A** to **B**) M detaches from actin filament on binding with ATP. (**C**) M hydrolyses ATP and binds with the actin filament in the form of M−ADP−Pi. (**D**) M performs the power stroke, associated with release of ADP and Pi from M. (**E**) After completion of power stroke, M remains bound with the actin filament. (**E** to **F**) On binding with next ATP, M detaches from the actin filament, and performs the recovery stroke to return to its neutral position. (**G**) M hydrolyses ATP and is ready to bind with the actin filament in the form of M−ADP−Pi. (**G** to **C**) M binds with the actin filament to restart the power stroke.

Figure 21 illustrates the sequence when ATP is applied to myosin heads in the actin–myosin filament mixture based on the above idea. Before ATP application, the myosin head (M, colored blue) forms rigor linkage with the actin filament at its neutral configuration (Figure 21A). On ATP application, M binds with ATP and detaches from the actin filament (Figure 21B). M then hydrolyzes ATP to form the complex M–ADP–Pi (charged-up state, colored red) and again binds with the actin filament at its neutral configuration (Figure 21C). Then M (red) performs the power stroke in the direction toward the myosin filament central bare region, to produce unitary actin–myosin filament sliding (C to D). After utilizing energy derived from ATP hydrolysis, M (blue) remains bound to the actin filament until the next ATP comes to bind with it (E). On binding with the next ATP, M (blue) detaches from the actin filament, and performs a recovery stroke to return to its neutral configuration (E to F), while hydrolyzing ATP to form M–ADP–Pi (F to G). M can then again bind with the actin filament (G to C) to repeat the power stroke. In muscle fibres at rest, M–ADP–Pi remains detached from the actin filament by the inhibitory effect of tropomyosin around actin filament (Figure 21G). Evidence has been presented [22,23,24] that M at the end of the power stroke (Figure 21E) takes a configuration which is definitely different from the well-known rigor configuration determined from extracted protein samples [25]. This issue is beyond the scope of this article.

It should be noted that our EC experiments showed that the recovery stroke in the direction away from the bare region takes place in two different conditions: (1) In the absence of actin filaments, the recovery stroke occurs from the myosin head neutral configuration (Figure 20); (2) in the presence of actin filaments, the recovery stroke occurs from myosin head post-power stroke configuration (Figure 21). Recovery stroke (2) is believed to takes place during the attachment-detachment cycle producing sliding between actin and myosin filaments, while recovery stroke (1) only takes place in the absence of actin filaments. We emphasize that the above interesting property of myosin heads in the myosin filaments was revealed only by our EC experiments. 

## 4. Freeze-Fracture Studies on Myosin Head Configurations

**Figure 22:**
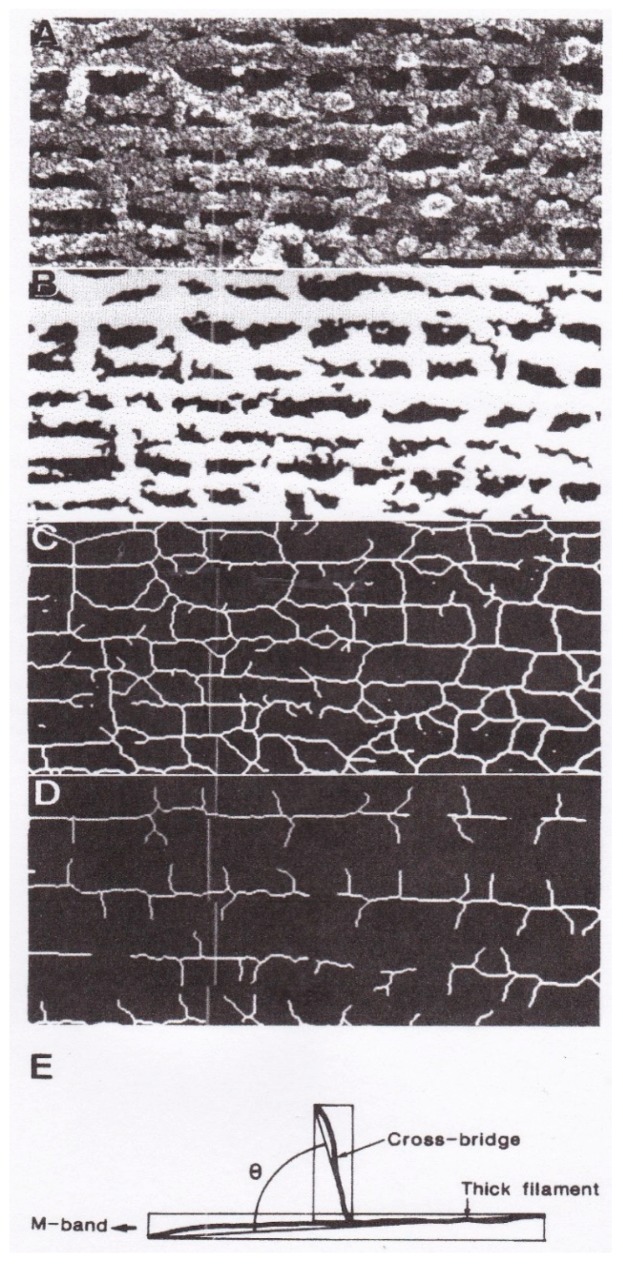
Measurement of myosin head angles to thin (actin) and thick (myosin) filaments using quick-freeze, deep-etch replica methods. Original replica image (**A**) was first converted into binary image (**B**), and further converted into the picture (**C**) after thinning process. After erasing lines of actin and myosin filaments as well as other unidentified structures, the picture (**D**), containing only lines of thin (actin) and thick (myosin) filaments and myosin heads was obtained. The cross-bridge (myosin head) angle to actin (or myosin) filaments was measured as the angle *θ* between the two diagonal lines as illustrated in (**E**). From Suzuki et al. [28].

Although the EC experiments are effective in revealing fundamental properties of myosin heads, the configuration of myosin heads at their neutral position is not clear. In this section, we will describe our freeze-fracture studies to give information about the myosin head configurations in relaxed, contracting and rigor muscle fibres. With the freeze-fracture method, it is possible to freeze the specimen surface in < 1 ms (Hauser, [26,27]). In our studies, single skinned muscle fibres were quickly frozen with liquid propane (~−190 °C), and freeze-etch replicas were prepared, in which adjacent single layers of actin and myosin filaments were exposed [28]. Figure 22 illustrates the procedures of measuring myosin head angles to the actin and myosin filament long axes with a digital image processor (Tospics II, Toshiba).

**Figure 23:**
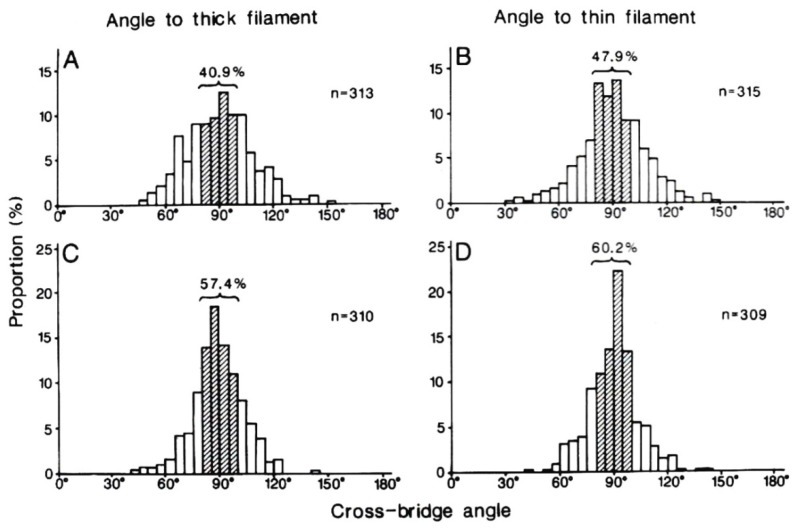
Histograms showing angular distribution of cross-bridges (myosin heads) to the thick (myosin) and the thin (actin) filaments in relaxed (**A**,**B**) and contracting (**C**,**D**) states. Number of measurements is given alongside each histogram. Histograms on the left side and on the right side shows myosin head angles to the thick (myosin) and to the thin (actin) filaments, respectively. From Suzuki et al. [28].

The results are summarized in Figure 23. In relaxed muscle fibres, the myosin head angle to the actin and myosin filaments showed a wide range of variation, reflecting myosin head thermal fluctuations with a peak at ~90°, i.e., perpendicular to the actin and myosin filaments (A and B). This result is consistent with the stability of myosin head position in the absence of ATP in the EC experiments, since in the EC experiments, the myosin head position is time-averaged over 0.1 s.

In contracting muscle fibres, the variation of myosin head angle was much smaller compared to relaxed fibres with a sharp peak at ~90° (C and D). These results can be taken to indicate that, in both the neural and the post-power stroke configurations, the myosin head CAD is perpendicular to the actin and myosin filaments. It follows from this that, during both the power and recovery strokes, the myosin head CAD remains rigid, and moves parallel to the actin and myosin filaments, keeping their configuration perpendicular to the filaments. In relaxed fibres, on the other hand, myosin heads are perpendicular to the filaments only at their neutral configuration; they are not perpendicular to the filaments when they are out of the neutral configuration, as indicated by the wide distribution of myosin head angle to the filaments (A and B).

## 5. Conclusions and Future Prospects

The EC experiments provide only means to directly visualize and record ATP-induced movement of individual myosin heads, since all other method can only study myosin head movement in concert based on various assumptions, which are not necessarily proved to be valid. On the basis of the results obtained from the EC experiments and the freeze-fracture studies on the myosin head configurations before and during the power and recovery strokes, myosin head movements can be summarized as follows. (1) In relaxed muscle, individual myosin heads fluctuate over a large distance around a definite neutral configuration; (2) In the neutral configuration, the myosin head CAD is perpendicular to the axis of actin and myosin filaments; (3) In the absence of actin filaments, individual myosin heads can perform a recovery stroke in the direction away from myosin filament central bare region (recovery stroke), without being guided by actin filaments; (4) The mean amplitude of the recovery stroke, in the absence of actin filaments, is the same (~6 nm) at both the distal and the proximal regions of the myosin head CAD; (5) In the actin–myosin filament mixture, in which only a small fraction of myosin heads can be activated by a limited amount of ATP applied, individual myosin heads perform a power stroke by stretching adjacent elastic structures; (6) The mean amplitude of the power stroke is 3.3 nm at the distal region and 2.5 nm at the proximal region of the myosin head CAD at the standard ionic strength, so that the myosin head CAD is oblique to both the actin and myosin filaments; (7) At low ionic strength, which is known to enhance Ca^2+^-activated isometric tension ~twofold, the mean amplitude of the power stroke increases to 5 nm at both the distal and the proximal regions of the myosin head CAD, so that the myosin head CAD is perpendicular to both the actin and myosin filaments during the course of the stroke. 

To summarize, when myosin heads hydrolyse ATP to form M–ADP–Pi (charged-up state), they perform a recovery stroke in the absence of actin filaments, and a power stroke in the presence of actin filaments; in both cases, they return to their neutral position after release of Pi and ADP. The EC experiments described above are extremely promising for future studies of the remaining mysteries in muscle contraction. For example, if the EC methods are coupled with those of laser flash photolysis of caged ATP [29] and time-resolved electron microscopy [30], we will be able to make a remarkable progress towards the full understanding of muscle contraction mechanisms. We are now planning to perform experiments with these methods.
